# Relationship Between Parental Communication Patterns and Self-Efficacy in Adolescents with Parental Substance Abuse

**Published:** 2020

**Authors:** Zeinab HEMATI, Samira ABBASI, Parastoo OUJIAN, Davood KIANI

**Affiliations:** 1 Nursing and Midwifery Care Research Center, Faculty of Nursing and Midwifery, Isfahan University of Medical Sciences, Isfahan, Iran.; 2 Department of Psychiatric Nursing, Faculty of Nursing and Midwifery, Isfahan University of Medical Sciences, Isfahan, Iran.; 3 Department of Pediatric Nursing, School of Nursing & Midwifery, Shahid Beheshti University of Medical Sciences, Tehran, Iran.; 4 Hajar hospital, Psychiatric ward, Shahrekord University of Medical Sciences, Shahrekord, Iran.

**Keywords:** Parental communication patterns, Self-efficacy, Adolescents, Substance abuse

## Abstract

**Objectives:**

Since an individual’s personality and behavior are mainly shaped in the family environment, parental substance abuse and its consequences can lead to confusion and instability in the family environment and reduce child-parent relationship. This study was conducted to investigate the relationship between different aspects of parental communication patterns and self-efficacy in adolescents with parental substance abuse.

**Materials & Methods:**

In this descriptive correlational study, a total of 100 adolescents with parental substance abuse were selected via random sampling. The data collection tools included the parental communication patterns scale and self-efficacy questionnaire. Data were analyzed in SPSS version 20, using linear regression analysis and Pearson’s correlation coefficient test.

**Results:**

The mean age of the adolescents was 14.5±2.5 years. Pearson’s correlation coefficient showed that the adolescents’ self-efficacy score was directly correlated with the score of conversation orientation dimension (a parental communication pattern) and inversely correlated with the dimension of conformity orientation (P<0.001).

**Conclusion:**

Development of educational programs to promote communication skills, positive family interactions, decision-making, and flexibility can change the unhealthy family environment into a healthy and productive one, which promotes self-efficacy beliefs in children.

## Introduction

Substance abuse imposes significant socioeconomic costs on communities. It has irreparable consequences for not only the individual, but also other people and the entire community. Since personality and behavior are mainly shaped in the family environment, substance abuse, both directly and indirectly, can destabilize moral and social foundations of families (1). Substance abuse and its consequences can be mirrored in family relationships, especially parent-child relationship (2, 3). Poor parental monitoring of children’s behaviors, parental conflicts, low quality of parent-child interaction, unstable family environment, and lack of intimacy and discipline are common in families with substance abuse (4). 

An unstable and confused family environment reduces children’s self-confidence and social skills, especially during adolescence, since adolescents are expected to deal with their problems effectively through empowerment and self-efficacy (5). In this regard, a previous study showed that conflict and incompatibility were inversely correlated with children’s self-efficacy (6, 7). Another study showed that parental participation and interaction in child rearing had significant effects on children’s self-efficacy (8). Furthermore, according to a study by Mohammadi et al., an unconventional family structure and parental incompetence in raising children can decrease the self-efficacy scores of adolescents with irresponsible parents (9).

Not only adolescents with parental substance abuse face numerous problems in their families, but also humiliating, angry, and indifferent behaviors of other people in the community impose an additional burden (10). Differences between adolescents raised in a cultural and informed environment with high moral values and those raised in an unhealthy environment arise from differences in these healthy and unhealthy environments (11). In this regard, Bendura argued that healthy families play an important role in the development of children’s self-efficacy beliefs through establishment of efficient interaction patterns, which influence children (12, 13). 

Considering the quality of family members’ interactions, communication content, activity type, communication milieu, and interfamilial interactions, McLeod and Cheffy presented two types of orientation to classify family interactions, namely conversation orientation and conformity orientation (14). Conformity orientation refers to an environment, which represents the family members’ conflicting attitudes, values, and beliefs. In contrast, conversation orientation addresses open and support conversations among family members so that all family members are encouraged to express their thoughts and emotions independently (15). However, there are no comprehensive studies on this subject. 

Because of the significant relationship between family interaction patterns and parental substance abuse, besides the significance of such relationships in adolescents’ self-efficacy beliefs in different aspects of life, we conducted this study to investigate the relationship between different aspects of parental communication patterns and self-efficacy in adolescents with parental substance abuse.

## Materials & Methods

This descriptive, correlational study was conducted from November 2014 to April 2016 on adolescents with parental substance abuse. The sample size was calculated to be 100, based on similar studies (power=90%; α=0.05; and dropout rate=20%) (16). 

The Research and Technology Deputy of Shahrekord University of Medical Sciences approved the study protocol, and permission was obtained from officials to conduct the study. Two centers were selected by lottery sampling. The researcher visited these two centers and explained the study objectives to the officials. A list of withdrawing parents with substance abuse was prepared in the centers, and 100 people were selected via random sampling. Then, the researcher contacted the parents, and if willing, the adolescents completed the questionnaires in the centers. If the parents did not consent to their children’s presence in the center, the questionnaires were handed to them to have their children complete the questionnaires and then return them to the center officials. 

To increase coordination and cooperation in completing the questionnaires, we asked the center officials for assistance (17). Before completing the questionnaires, if the adolescent was above 18 years, the researcher requested him/her to consent to participation in the study. On the other hand, if he/she was under 18 years, the parents were asked to give their consent. Also, the researchers gave explanations on how to complete the questionnaires. This study is part of a research project, approved by the Research and Technology Deputy of Shahrekord University of Medical Sciences (1392-01-83-1661). Relevant authorities approved the study protocol.

The inclusion criteria were as follows: 1) parental (father or mother) withdrawal of substance abuse; 2) age range of 11-21 years; 3) lack of any disease or mental retardation; 4) mental health and full consciousness (having no critical or emergency conditions during the study); 5) lack of stressful events, such as death of close relatives within the past month; 6) living with parents; and 7) not having divorced parents. The exclusion criterion was unwillingness to participate in the study. 

For data collection, a demographic questionnaire, parental communication patterns scale, and Schwarzer and Jerusalem self-efficacy questionnaire were used. The revised parental communication patterns scale, developed by Fitzpatrick et al., contains 26 items, rated on a five-point Likert scale, ranging from absolutely agree (score 5) to absolutely disagree (score 1) to investigate two dimensions of conversation orientation (15 items) and conformity orientation (11 items). Farahati et al. reported a Cronbach’s alpha coefficient of 82% for the conversation orientation dimension and 80% for conformity orientation. The validity of the scale was measured to be 6.48 for conversation orientation and 3.26 for conformity orientation according to factor analysis and internal consistency (18, 19). 

Moreover, Schwarzer and Jerusalem self-efficacy scale consists of 10 items, rated on a four-point Likert scale. This scale reflects an optimistic self-belief that one can perform a novel or difficult task or cope with different challenging situations in various domains of human functioning (20). Higher scores represent higher levels of self-efficacy. This questionnaire has been used in a number of studies, with Cronbach’s alpha coefficients of 75-90% (21, 22). Data were analyzed in SPSS version 20, using linear regression analysis and Pearson’s correlation coefficient test. 

## Results

According to the results, the mean age of the adolescents was 14.5±2.5 years. The majority of adolescents (51%) were male. Also, most fathers (67%) were self-employed, and most mothers (80%) were housewives (Table 1). 

Pearson’s correlation coefficient showed that the adolescents’ self-efficacy scores were directly correlated with the score of conversation orientation dimension and inversely correlated with the conformity orientation dimension as a parental communication pattern (P<0.001) (Table 2).

Among two parental communication patterns, effect of conformity orientation was greater on the adolescents’ score of self-efficacy. Conformity orientation was inversely correlated with the score of self-efficacy; in other words, it reduced self-efficacy. However, the adolescents’ score of self-efficacy improved with an increase in the score of conversation orientation (Table 3). Meanwhile, linear regression analysis of the adolescents’ self-efficacy scores (Y), according to parental communication patterns of conversation orientation (x_1_) and conformity orientation (x_2_), indicated the following results: Y= 29+0.126X_1_-0.183X_2_.

**Table 1:** Demographic characteristics of the participants

**Demographic **	**variables**
Sex, N (%)	
Male Female	51 (51) 49 (49)
Child’s educational level, N (%)	
Elementary school Secondary school High school	19 (19) 31 (31) 50 (50)
Father’s educational level, N (%)	
Elementary school Secondary school Diploma University degree	16 (16) 8 (8) 37 (37) 39 (39)
Mother’s education, N (%)	
Illiterate Elementary school Secondary school Diploma University degree	13 (13) 15 (15) 13 (13) 38 (38) 21 (21)
Father’s employment status, N (%)	
Self-employed Employed Others	67 (67) 32 (32) 1(1)
Mother’s employment status, N (%)	
Housekeeper Employed	80 (80) 20 (20)

**Table 2:** Pearson’s correlation coefficients of parental communication patterns and self-efficacy scores

Parental communication dimension	Self-efficacy score	
	r	P-value
Conversation orientation	0.402	<0.001
Conformity orientation	-0.424	<0.001

**Table 3:** Linear regression analysis for prediction of adolescents’ scores of self-efficacy with respect to the scores of parental communication patterns

Parental communication dimension	Self-efficacy score		
	B	Beta	P-value
Conversation orientation	0.126	0.269	<0.001
Conformity orientation	-0.183	-0.308	<0.001

## Discussion

The present study was conducted to investigate the relationship between different aspects of parental communication patterns and self-efficacy in adolescents with parental substance abuse. Based on the results, the adolescents’ self-efficacy was directly correlated with the score of conversation orientation and inversely correlated with the score of conformity orientation as a parental communication pattern.

In this regard, Dehghanizadeh et al. showed that family environment could promote self-efficacy and academic vitality of students if they enjoyed a family environment where they could establish open communications and were encouraged to express their emotions, beliefs, and ideas, which were taken into account by the parents in the process of decision-making (15). Furthermore, Abolghasemi et al. reported that life skills education promoted flexible decision-taking in unpredicted circumstances, improved self-efficacy among spouses of people with substance abuse through empowerment, increased the feeling of responsibility among family members, and encouraged them to participate in certain activities, such as effective discussion and debate (23). 

Moreover, Fan et al. and Ugoji examined the association of children’s self-efficacy with intimacy among family members, parental advice for children, child-rearing approaches, and family communication patterns (24, 25). Another study showed that adolescents with poor family relationships are more likely to have low personal competencies (26). To explain these findings, we can argue that open and positive child-parent relationship can increase intimacy and closeness among family members and help resolve problems and difficulties in a flexible and intimate environment. 

In families with strong conversation orientation, hierarchy is less important, and all family members can express their beliefs, ideas, and attitudes openly. In these environments, parents respect each other and recognize the adolescents’ individuality and independence. In such environments, adolescents can feel independent and courageous enough to express their emotions and opinions even if they disagree with their parents. Discussion and exchange of ideas happen in a logical manner, and adolescents are not questioned for their ideas. As a result, they experience higher levels of control over life events and self-efficacy. Indeed, a healthy family can cope with and solve problems effectively and make appropriate decisions in dealing with children’s problems. Adolescents in such families feel more confident and self-efficacious in dealing with challenges and are less likely to become disappointed due to their great assiduity and effort (27). 

According to the findings of the present study, conformity orientation was associated with reduced self-efficacy in adolescents, while scores of self-efficacy increased as family conversation increased. In this regard, Sepehri et al. found that families, which insist on children’s conformity to beliefs and attitudes, obedience to parents, hierarchy, and limited communication and interaction, raise children who are likely to exhibit lower resilience to difficulties due to certain behaviors and thoughts, such as hostility and evasion of problems (10). Dehghanizadeh et al. indicated that parental conformity orientation, unlike conversation orientation, could not lead to the development of academic vitality in students (15). 

Another study reported a significant inverse correlation between parental conformity pattern and students’ self-concept (28). Considering the emphasis of conformity orientation on convergence of attitudes and beliefs, avoidance of conflict, and dependence of family members on each other, adolescents consider it essential to cope with family conditions and show unquestioning obedience. Behaviors of these adolescents may be based on their parents’ expectations; therefore, their personality is not developed, and they fail to exhibit suitable behaviors in different environments.

The most important limitation of the present study was that some parents were unwilling to have their children complete the questionnaires. However, the researcher mitigated this limitation by asking the clinic experts to negotiate and communicate with these parents so that they would consent to their children’s participation in the study.

## Conclusions

**In Conclusion, **Based on the present results, self-efficacy is directly associated with the dimension of conversation orientation as a parental communication pattern. Therefore, development of educational programs to promote communication skills, positive family interactions, effective decision-making, and flexibility can change an unhealthy family environment into a healthy and productive one, where self-efficacy beliefs are developed in children. In addition, since such beliefs can be learnt, we can teach them to adolescents, particularly those with substance-dependent parents, with the assistance of school advisors. A few clinical trials have been performed to determine interventions, which may affect the adolescents’ self-efficacy and parental communication patterns. Therefore, in future interventional studies, it is important to identify parental communication patterns and self-efficacy of adolescents with parental substance abuse. 

## Author`s Contribution

Hemati Z: Substantial contributions to the conception or design of the work; or the acquisition, analysis, or interpretation of data for the work, Drafting the work or revising it critically for important intellectual content. Abbasi S: wrote the first draft, revised of this manuscript. Oujian P: contributed to the study design and helped to the edition of the article kiani D: supervised the study, revised the manuscript and final approval of the version to be published**.**
